# P10-10 Technology-supported exercise may increase self-reported health status in people with residual movement impairments after neurological event

**DOI:** 10.1093/eurpub/ckac095.149

**Published:** 2022-08-29

**Authors:** Eveline Graf, Dino De Bon, Daniel Zutter, Markus Wirz

**Affiliations:** 1 School of Health Sciences, Zurich University of Applied Sciences, Winterthur, Switzerland; 2 VAMED Management and Service Switzerland AG, Zurich, Switzerland; 3 Rehaklinik Zihlschlacht, Zihlschlacht, Switzerland

**Keywords:** technology-supported exercise, gait impairment, neurological, training

## Abstract

**Background:**

After a neurological event (e.g. stroke) people are often restricted to a sedentary lifestyle due to chronic impairment. Their need for assistance to perform any form of physical activity (PA) is one factor that limits their PA and results in a negative impact on health. Rehabilitation technology, designed for gait rehabilitation during sub-acute rehabilitation, may be used to achieve the recommended levels of PA in people with gait impairments. However, it is unknown if there are effects on the quality of life resulting from such exercise regimen. Therefore, the aim of this study was to determine the effect of technology-supported exercise (TSE) on self-reported health status.

**Methods:**

Twelve people with severe residual gait impairment after a neurological event participated in the study. They performed TSE (with Lokomat, Andago or C-Mill) for three months (M3) with a minimum of 10 trainings per month. At baseline and M3, the following questionnaires were answered: EQ-5D-3L, WHODAS 2.0, patient global impression of scale (PGIC, only at M3). Wilcoxon-signed-rank-test was used to test for statistically significant differences between start and M3 (p > 0.05).

**Results:**

The median EQ-5D Visual Analog Scale (VAS) score at baseline was 60, which is lower than the population mean. The VAS of the EQ-5D showed significant improvements (median: 7.5 points). The median of the PGIC was ‘minimally improved’ while all other outcomes (EQ-5D domains and WHODAS) remained constant.

**Conclusion:**

Prior to TSE, the self-rated health status was low. There is evidence, that people with residual gait impairment may benefit from continuous TSE by improving health status, as has been represented by the improvement of the EQ-5D VAS score. Most chosen questionnaires may not be sensitive enough to detect subtle changes, indicating that for this population more sensitive instruments may be needed. However, future research investigating the sensitivity of the questionnaires is advisable. In addition, the training period of three months might be too short to be effective. This study is still ongoing with the training period extended to six months and additional participants included which will provide more data about the effect of TSE on self-reported health status.

